# Paper in an Electronic World – the Utility of an Integrated Treatment Booklet for the Safe Provision of Electroconvulsive Therapy (ECT) in a Regional Australian Mental Health Service (MHS)

**DOI:** 10.1192/bjo.2025.10677

**Published:** 2025-06-20

**Authors:** Leo Smith, Andrew Robertson

**Affiliations:** South West Healthcare, Warrnambool, Australia

## Abstract

**Aims:** It is incumbent upon psychiatrists to manage cognitive and physical health sequelae during a course of ECT. Monitoring post-seizure orientation and the stability of Montreal Cognitive Assessments (MoCAs) over time allows for dynamic changes to modality, frequency and energy settings in order to minimise side effects. Our service hypothesised that disparate electronic forms actually hindered this process and therefore conducted an audit.

An integrated paper-based treatment booklet for use within the ECT suite, with all forms bound together, was piloted as the quality improvement intervention. A new post-seizure orientation tool was also used.

**Methods:** The setting was South West Healthcare (SWH), Warrnambool, Australia. Standards were set a priori according to ECT guidelines from the Victorian Office of the Chief Psychiatrist and the Royal Australian and New Zealand College of Psychiatrists, with 80% compliance targeted. At a minimum, patients needed baseline bloods (full blood count; urea/electrolytes/creatinine), electrocardiograph, physical examination and MoCA, then physical/MoCA after every third treatment. Furthermore, a comment on orientation in the recovery suite after each treatment was required to meet standard.

Files were selected by 26/06/23 (cut-off date), capturing all ECT patients in the 6 months prior. 15 patients were identified, a combination of acute/completed and acute-continuation/maintenance ECT. Records, both paper and electronic, were audited against standards over 4 consecutive weeks by the authors. After the results were reviewed, the integrated treatment booklet (designed by the lead author) and post-ECT orientation questionnaire (licensed from the University of New South Wales) were introduced into clinical practice.

The audit cycle was completed a year later, with files selected by 30/08/24, capped at 20 patients and capturing all those who had had ECT since the pilot began.

**Results:** The baseline standard during the initial audit was generally met: bloods (79%), ECG (86%), physical (64%), MoCA (86%). However, the standard was not achieved once ECT commenced: physicals every 3rd treatment (60%), MoCAs (49%). Orientation status was documented in 90% of treatments.

During the post-intervention re-audit, compliance had vastly improved: baseline bloods, ECG, physical and MoCAs (100%); objective orientation scores (99%); ongoing physicals (76%)/MoCAs (72%).

**Conclusion:** Whilst not quite reaching the 80% compliance target overall, the integrated treatment booklet, with monitoring of re-orientation, significantly improved the cognitive/physical health tracking of patients undergoing ECT at SWH. With further operational change, full compliance is anticipated in the future. Returning to paper was universally supported by psychiatrists and managers, with clinical utility demonstrated within the ECT suite.

